# Influencing Decisions of Value in Health: A Response to Recent Commentaries

**DOI:** 10.15171/ijhpm.2018.116

**Published:** 2018-11-24

**Authors:** Iestyn Williams, Hilary Brown, Paul Healy

**Affiliations:** ^1^Health Services Management Centre, University of Birmingham, Birmingham, UK.; ^2^NHS Confederation, London, UK.

We are delighted to have received three responses to our recent paper ‘contextual factors influencing cost and quality decisions in health and care’^1^ and would like to revisit a few of the many interesting points raised.

In their commentary, Stuart Peacock and Colene Bentley focus on priority setting and disinvestment decision-making.^2^ They note that ‘it is quite evident that disinvestment decisions are often treated fundamentally differently from adoption and coverage decisions.’ This very much matches our own experience.^3^ Indeed, the comparison illustrates the dynamic nature of contextual influence, whereby decision characteristics (eg, intervention *removal* versus intervention *adoption*) trigger distinct responses from within the organisational and wider context. This means that these contextual influencers should not be portrayed as static or fixed, and this brings us to Kristine Bærøe’s concerns about our conceptualisation of context. We acknowledge her claim that we wish (in our words!) to have our cake and eat it; in other words that we combine an interpretivist approach on the one hand, with an aspiration to codification and measurement on the other. We believe that this is a productive tension in health services research and, although we are happy to revisit these weighty and timeworn debates outside of these pages, we have no desire to breathe new life into old paradigm wars.

Peacock and Bentley take up our normative claim (which, we concede to Bærøe, is out of step with an otherwise analytical article), that external engagement should be greater where decisions of value are on a larger scale. They argue for deliberative approaches and again we agree. Distinctions between ‘formal’ and ‘arbitrary’ influencers,^4^ and between front and backstage decision making,^5^ are extremely useful for research in this field and we will incorporate them in future. We support Michael Calnan’s call for ethnographic observation in this research area, and indeed our own work on the use by NICE of economic evaluation appears to support his conclusions.^6,7^ Finally, we welcome Calnan’s reminder of the irreducibly social nature of decision making, and of the mediating role played by trust.

We conclude by restating our interest in the relatively neglected areas of what we have termed ‘technical’ decisions, and perhaps these are less amenable to the types of analyses that we describe in the paper and in this response. However, decisions over, for example, organisational restructuring, capital spending and workforce –are no less important and certainly no less contextually embedded than allocative decisions.

## Ethical issues

Not applicable.

## Competing interests

Authors declare that they have no competing interests.

## Authors’ contributions

All authors were involved in drafting and approval of this response.

## Authors’ affiliations

^1^Health Services Management Centre, University of Birmingham, Birmingham, UK. ^2^NHS Confederation, London, UK.
